# Estimation of the dose of hyperbaric bupivacaine for spinal anaesthesia for emergency caesarean section in an achondroplastic dwarf

**DOI:** 10.4103/0019-5049.71030

**Published:** 2010

**Authors:** Tanvir Samra, Sujata Sharma

**Affiliations:** Department of Anaesthesia and Intensive Care, Dr Ram Manohar Lohia Hospital, Baba Kharak Singh Marg, Connaught Place, New Delhi - 110 001, India

Sir,

Achondroplasia is the most common condition associated with short-limbed dwarfism. Anaesthetic management is challenging as patients are at increased risk of airway complications if administered general anaesthesia due to limited neck extension, large head, large tongue and narrowed nasal, oral, tracheal and pharyngeal airway, failure of neuraxial anaesthesia because of thoracolumbar deformity and spinal canal stenosis[1] leading to controversy over the dose requirement for spinal anaesthesia. We describe the successful administration of spinal anaesthesia and the rationale for the dose of hyperbaric bupivacaine injected in an obese achondroplasic parturient.

A 24–year-old primigravida with cephalopelvic disproportion underwent emergency caesarean section at 38 + 2 weeks of gestation in view of foetal distress. Her height was 126 cm, weight was 64 kg (prepregnancy weight of 55 kg) and her body mass index (BMI) was 34.6 kg/m^2^ with lumbar lordosis but normal thoracic spine. Her Malampatti score was 3, with normal interincisor and thyromental distance.

Antacid prophylaxis was administered, preloading with 500 ml of normal saline done and pulse oximetry, electrocardiogram (ECG) and an automatic blood pressure cuff attached for monitoring. 1.6 ml of 0.5% of hyperbaric bupivacaine (8 mg) was administered in the L3-L4 intervertebral space using a 26 G Quinckes needle. Sensory blockade extended to T4 and the patient maintained haemodynamic stability intraoperatively.

The factors considered when calculating the dose of hyperbaric bupivacaine in our patient were height, weight, spinal anatomy and pregnancy, as these are important determinants of intensity and duration of spinal block. The minimum effective dose of intrathecal bupivacaine providing effective spinal block in 95% of the women undergoing caesarean section is 0.06 mg/cm height[2] and, based on this for our patient (height 126 cm), the dose of bupivacaine was 7.56 mg or, approximately, 8 mg (1.6 ml of 0.5%). Achondroplasia is associated with spinal canal stenosis and, as proposed in a similar case report by Ravenscroft *et al*.,[3] a 30% reduction in the dose of intrathecal drugs should be carried out in parturients with achondroplasia. But, in our case, no reduction in dose was performed as a magnetic resonance imaging of the spine did not show any evidence of spinal canal stenosis. It has been suggested by imaging that the lumbosacral cerebrospinal fluid volume varies inversely with BMI. But, in spite of this, clinical studies by Norris[4] demonstrated that the dose of intrathecal bupivacaine for caesarean delivery is similar in obese and normal weight women.

Previous studies have used 1.3 ml of 0.5% hyperbaric bupivacaine with 10 mg of fentanyl,[3] but these studies reported a transient decrease in blood pressure, or 1 ml of 0.5% hyperbaric bupivacaine with 10 mg of fentanyl.[5] But, none of the studies gave any rationale for drug dosage of intrathecal bupivacaine used.

The probability of failed spinal is higher in achondroplastic patients but, still, parturients undergoing caesarean section should not be denied regional anaesthesia. The dose of intrathecal bupivacaine injected must be based on the height of the patient, with corrections made for the presence of spinal canal stenosis. However, no reduction in the dose is needed for obesity.
